# Sense of Coherence and Connectedness to Nature as Predictors of Motivation for Practicing Karate

**DOI:** 10.3390/ijerph16142483

**Published:** 2019-07-12

**Authors:** Mariusz Lipowski, Daniel Krokosz, Ariadna Łada, Miroslav Sližik, Marcin Pasek

**Affiliations:** 1 Faculty of Tourism and Recreation, Gdansk University of Physical Education and Sport, 80-336 Gdansk, Poland; 2 Institute of Psychology, University of Gdansk, 80-309 Gdansk, Poland; 3 Department of Physical Education and Sports, Matej Bel University, 974 01 Banská Bystrica, Slovakia

**Keywords:** connectedness to nature, sense of coherence, mental health, karate, sport

## Abstract

*Background*: Physical activity yields exceptionally positive results when it takes place out in the open air, in contact with nature. Both contact with nature and practicing sport constitute a certain kind of philosophy of life and living by its rules plays a vital role in positive mental health―understood as maintaining a high sense of coherence. Martial arts are examples of sports that are rooted in a philosophy. The goal of this study was to explore the links between a sense of coherence and connectedness to nature in the context of motivations for practicing karate. *Methods*: A total of 127 practitioners of karate were examined using the Inventory of Physical Activity Objectives (IPAO), the Sense of Coherence Questionnaire, and the Connectedness to Nature Scale. *Results*: The most important objective for women training karate was a fit, shapely body, and for men the most important objective was physical fitness. Connectedness to nature had the strongest positive relationship with the measure of physical fitness (in both genders). A sense of comprehensibility increased men’s motivational conflict, whereas, in women, this IPAO dimension was positively related to feeling emotionally connected to the natural world. Connectedness to nature was related to motivational conflict positively in women and negatively in men. Feeling emotionally connected to the natural world correlated with a sense of comprehensibility, a sense of manageability, and a sense of meaningfulness. Sense of comprehensibility and sense of meaningfulness tended to increase with age. *Conclusions*: Understanding both the natural environment and the utility of setting sport-related goals led to increasing one’s efforts on the way to both successes and defeats, and, most of all, overcoming one’s weaknesses.

## 1. Introduction

Regular engagement in physical activity (especially outdoors) plays an important role in shaping one’s physical and mental health [1,2]. Despite many informational and preventive campaigns, lifestyle changes have led to a pandemic of physical inactivity [3]. This is associated with spending extended periods of time in closed spaces; many sources suggest that even just being in the natural environment may have a positive influence on humans’ physical and mental health [4]. Meta-analyses indicate that this may have a significant influence on enhancing well-being and affect, providing a sense of distance from daily problems and developing a sense of belonging to the surrounding world [5,6]. Connectedness to nature is a measure of an individual’s levels of feeling emotionally connected to the natural world and is used in the realm of social and environmental psychology [7]. Mental presence in the environment or being in the here-and-now is associated with the recent trend of mindfulness, which is popular especially in psychotherapy and sport psychology. This approach stems from an interest in Eastern philosophy, especially Zen Buddhism [8]. Mindfulness practices, such as conscious breathing, being in the here-and-now, and non-judgmental observation, are also often used in Eastern martial arts training [9]. Martial arts belong to a particular group of sport disciplines which are very rarely practiced outdoors, especially when it comes to tournaments and competitions. Therefore, carrying out at least some parts of the training process in natural open spaces is justified, which would make it possible to use natural environmental elements as part of the training. The idea of harmonizing the training of martial arts with nature is also hidden in the well-known Japanese calligraphy of old warriors under the name “Sekai-dojo”―*My “dojo“ (my gym), is my universe.* All budo (martial art) students eventually realize that their behavior has changed and has been greatly influenced by the martial art discipline they practice. They become more conscious, braver, more careful, more attentive, and more respectful of others. They are able to better acquire natural principles in their philosophical context. They are able to exercise greater willpower, frankness, and generosity. This is followed by the next step: Introducing these virtues into ordinary life outside the dojo (gym). After this happens, the whole world becomes a large, wide dojo. Research shows [10] that this attitude is particularly visible in athletes who engage in traditional forms of karate, which place an emphasis on values. Outdoor physical activity also provides more opportunities to achieve states of mental relaxation, which is so important in fighting sports that some even consider it fundamental. During such a training process, three areas overlap: Physical activity, relaxation, and ecological contemplation [11]. Hence, one might ask whether these spheres are interrelated. It is debatable, however, that physical activity goes hand-in-hand with forming ecological attitudes. It is important [8] that, through contact with nature, one achieves relaxation [12], which can effectively motivate one to take up physical activity [13] or, at the very least, improve one’s well-being [14]. Thus, these relationships are worth analyzing in terms of the homeostasis maintained by people who are physically active, in particular those who practice combat sports.

Mental health, somatic health (indirectly), and the entirety of psychophysical functioning depend on, among other things, interpersonal communication [15,16], physical activity, and a person’s direct relationships with their natural surroundings. These factors can have many benefits for one’s mental well-being [17]. The peculiar charm of contact with nature can effectively prepare one to better organize one’s free time by forming habits [9] in the areas of leisure and health [18]. The literature even mentions *vitamin G* [19], a term derived from the English word *green*. It is meant to refer to contact with verdant areas which surround humans and shape their attitudes, sense of well-being [20], and self-esteem [21], while at the same time diminishing aggression and aggression-related crimes [22]. Spending time in the natural environment is likely to yield a stronger sense of connectedness to it [23] and can result in going out in the open air more frequently [24]. Yet, positive experiences are often the only benefit attributed to sports and recreation camps―building healthy attitudes toward nature is rarely mentioned [25].

In some studies, the advantages of exercising out in the open (as opposed to practicing in confined spaces) were found to be debatable and were mentioned as a research question rather than a result [16,25]. On the other hand, some studies claim that open field activities are superior, suggesting this in their titles and confirming it with substantive findings [26]. At any rate, we can theorize that, regardless of the type of activity, both kinds of practices are driven by similar motivations.

To date, studies which explore such motivations rarely mention the benefits provided by the natural environment, such as silence, toned-down colors (which positively influence the functioning of one’s nervous system), fresh air, and scenic aspects of the landscape [27]. Athletes more often point to motives like feeling pleasure or a sense of challenge, while those who engage in physical activity recreationally are motivated by goals of weight control and stress reduction [28,29].

Many studies examining motivations for practicing combat sports have focused on particular disciplines [30], specific groups [31], or differences in how much time one devotes to such sports [32]. At the same time, it has been suggested that there are three main groups of driving forces which motivate practitioners to be active: Physical and psychological, intellectual and emotional, and transcendental motivations [33]. Apart from the well-established influence on an individual’s mental health [34,35], a recurring theme in these studies is the belief that the activity in question facilitates bringing together the spheres of spirit, body, and mind―this motive has already been found to be fundamental in some studies [36]. After all, martial arts naturally combine physical activity, meditation, and breath control (dominated by diaphragmatic breathing), allowing for building self-discipline as well as developing ambition and respect for one’s opponent. In this way, martial arts can be a specific method for improving one’s mental health, one that emphasizes relaxation, self-esteem, and psychomotor coordination [25].

The nature of fighting sports results in a considerable difference in the numbers of male and female participants, to the point that these sports are sometimes regarded as male-only activities [37,38]. This poses a problem regarding the justification of participant categorization by sex when exploring variables. A lot of data [39] on the differences between sexes regarding motivations for physical activity, a sense of coherence, and connectedness to nature suggest that this is justified [40]. 

One might theorize that both contact with nature and practicing sports make up a certain kind of philosophy of life and that living by its rules plays a vital role in staying healthy, understood as maintaining a high sense of coherence. The fundamental assumption in this life orientation is that, since most of the time individuals are unable to reduce stress directly, their attitude to the world should be shaped in such a way that will allow them to act creatively in it. This is highly conducive to forming health-promoting attitudes [41,42]. This generalized cognitive–emotional orientation to life is made up of three distinct components: Comprehensibility, manageability, and meaningfulness [43]. Comprehensibility is an individual’s ability to cognitively encompass the surroundings that currently affect them. Manageability is defined as feeling capable of dealing with one’s surroundings; this feeling comes from being aware of having some specific tools at one’s disposal. Finally, meaningfulness is being sure that engaging and investing in one’s life is both reasonable and desirable and through this lens, stressful situations may be viewed as challenges rather than threats. 

Based on the preceding discussion, we wish to explore the following research question: Is there any connection between a sense of coherence and connectedness to nature in terms of the motivational function of one’s objective when practicing karate? Finding the answer to this question can shed a new light on understanding the relationships between environment, mental health, and self-regulatory functions, such as goal setting and motivation.

The following hypotheses were tested:

1. A sense of coherence and feeling emotionally connected to the natural world are related to both the formation of objectives for practicing karate and the motivational function of these objectives.

2. The relationship between sense of coherence and feeling emotionally connected to the natural world is different for male and female participants.

## 2. Materials and Methods

### 2.1. Participants

A total of 154 experienced karate practitioners were invited to participate in the research. Although 132 positive responses were received, 5 answer sheets were uncompleted. The final group consisted of *n* = 127 karate practitioners (42 women, 85 men; age range was 18‒65, M_age_ = 31.66, SD_age_ = 11.59, no gender differences). Each participant attended a course at one of the following clubs in Poland: Champion in Gdańsk, Zanshin Karate in Gdynia, or Kyokushin Karate in Tczew. The clubs were chosen randomly from a database of Karate clubs in the Pomeranian Voivodeship in Poland. Only experienced practitioners who had been practicing karate for at least three years and who had reached a minimum of 5 Kyu in Shotokan (blue belt student degree) or 4 Kyu in Kyokushin (green belt student degree) were included in the study. 

### 2.2. Procedure

The protocol of this study was approved by the Ethics Board for Research Projects at the Institute of Psychology, University of Gdansk, Poland (decision no. 8/2014). Subject sampling was purposive. Prior to the study, written informed consent was obtained from the participants and they were informed that they could discontinue their participation at any time without repercussions. Respondents were given three days to complete the questionnaire pack, which was then returned to one of the investigators. All participants received the Inventory of Physical Activity Objectives [44], the Sense of Coherence questionnaire [45], and the Connectedness to Nature Scale [8]. Having agreed to participate, all subjects returned the completed questionnaire forms. The data used for this study were part of a larger survey and the questionnaires used in this study took around 30 min to complete.

### 2.3. The Inventory of Physical Activity Objectives (IPAO)

The original version of The Inventory of Physical Activity Objectives by Lipowski and Zaleski [44] was used in order to determine the level of involvement in practicing karate and to measure the motivational function of one’s goal in this discipline. Four scales of goal-oriented behaviors associated with practicing karate were distinguished in the test: (a) Motivational value, (b) time management, (c) persistence in action, and (d) motivational conflict. Respondents could choose multiple objectives underlying their karate training. Motivational value refers to the extent to which the objective influences the actions undertaken by an individual; time management refers to the level of focus on planning, arranging, and organizing time for karate; persistence in action describes efficiency and persistence of action, as well as the ability to deal with adversities; finally, motivational conflict describes to what extent karate objectives are in conflict with other objectives. Each participant responded to 18 items, selecting to what extent they agreed with a given statement (on a Likert scale: 1 = “I do not agree at all” to 5 = “I fully agree”). The Inventory of Physical Activity Objectives also included 12 objectives which were accompanied by a Likert scale (1‒5); the respondent was asked to assess the importance of the listed objectives, where 1 stood for completely unimportant and 5 for very important. In addition to measuring the attitude of the subjects toward particular goals, the total score (all Likert-scale answers added up) was also tracked. The final score indicated the importance of heterogeneity of the objectives one sets. The reliability coefficient α of IPAO reached 0.78.

### 2.4. The Sense of Coherence Questionnaire (SOC-29)

The SOC-29 [45,46], in its Polish adaptation [47], contained 29 questions, 11 of which related to the sense of comprehensibility subscale (i.e.,“When you talk with people you feel that they don’t understand you”), 10 to the sense of manageability subscale (i.e., ” Do you have the feeling that you are treated unfairly”), and 8 related to the sense of meaningfulness subscale (i.e., ”Life is…very interesting―1/monotonous―7). Respondents provided self-reported ratings on a 7-point Likert-type scale ranging from 1 to 7. Please note that the issue of SOC-29′s factor structure has not been settled in terms of the 3-factor construction of the sense of coherence. In this study, Pasikowski’s [48] standpoint was adopted, according to which it is advisable, even necessary, to calculate not only the total score for sense of coherence, but also for its components. The evaluation of the Polish version of the SOC-29 questionnaire showed very high reliability of the tool. Indices of internal consistency, calculated using the half-method with the Spearman–Brown correction, were as follows: 0.92 for sense of coherence; 0.78 for sense of intelligibility; 0.72 for sense of resourcefulness; and 0.68 for sense of sense. Cronbach’s alpha was 0.78 [48].

### 2.5. The Connectedness to Nature Scale (CNS)

The Connectedness to Nature Scale [8] provided a measure of an individual’s trait levels of feeling emotionally connected to the natural world. It was designed to measure the extent to which participants generally felt part of the natural world and emotionally connected to it. This measure consisted of 14 items rated on a 5-point Likert scale (1 = strongly disagree, 5 = strongly agree). The instructions for respondents read: “Please answer each of these questions in terms of how you generally feel. There are no right or wrong answers. Using the following scale, in the space provided next to each question, simply state as honestly and candidly as you can what you are presently experiencing.” Items 4, 12, and 13 were reverse-scored. The scores were then added up and the total ranged from 14 to 70. Higher scores reflected a higher degree of affective connectedness to nature.

### 2.6. Statistical Analyses

Statistical analyses were conducted with the Polish version of STATISTICA 12 (TIBCO, Palo Alto, CA, USA), and included general linear models, multiple regression, Pearson’s *r* correlation, and indicator variables in regression. There were two steps in the empirical data analysis process. First, the motivational function of the practicing karate objective, sense of coherence, and connectedness to nature were profiled. Then, the analysis of the relationships between sense of coherence and levels of feeling emotionally connected to the natural world vis-à-vis the motivational function of the training goal was conducted. Data gathered from males and females were analyzed separately. The significance threshold was set at 0.05. Multiple regression was used to test the first hypothesis and indicator variables in regression were used to test the second hypothesis.

## 3. Results

The first step of the analyses was to assess the mean values of and gender differences in the investigated variables. Analysis of the importance of motivations for doing the sport showed that, on average, the most important goal for women was well-being, while for men it was physical fitness. A detailed summary of the means of the importance of goals is presented in Table 1.

Next we examined which goal was selected most often by the athletes as the one which most motivates them to do karate. Women most frequently selected having a fit, shapely body (23.81%), while men most frequently selected physical fitness (41.18%). No one chose the following objectives as the most important: Company of others, fulfilling the need for activity, or promoting physical activity.

**Figure 1:**
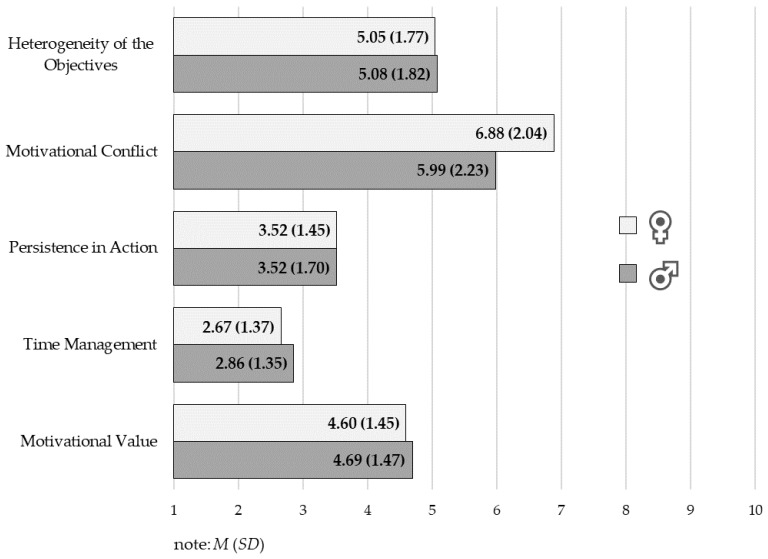
Motivation function of the goal in women and men practicing karate.

An analysis of the motivational function of the goal was done for the most motivating goal for each of the participants. There was only one difference found between men and women; this regarded motivational conflict, higher levels of which were observed among women (*M*_♀_ = 7.88, *SD*_♀_ = 2.04, *M*_♂_ = 6.99, *SD*_♂_ = 0.23, *t* = 2.18, *p* = 0.031). Results in sten scores are presented in Figure 1.

No differences between women and men were observed in levels of sense of coherence or connectedness to nature. Mean results for these variables are presented in Table 2.

Correlation analysis confirmed the assumption that, among both women and men, levels of feeling emotionally connected to the natural world correlated with sense of comprehensibility (*r*_♀_ = 0.38, *p*_♀_ = 0.014, *r*_♂_ = 0.37, *p*_♂_ = 0.001), sense of manageability (*r*_♀_ = 0.41, *p*_♀_ = 0.008, *r*_♂_ = 0.36, *p*_♂_ = 0.001), and sense of meaningfulness (*r*_♀_ = 0.49, *p*_♀_ = 0.001, *r*_♂_ = 0.36, *p*_♂_ = 0.001). For both sexes, sense of comprehensibility (*r*_♀_ = 0.46, *p*_♀_ = 0.002, *r*_♂_ = 0.44, *p*_♂_ < 0.001) and sense of meaningfulness (*r*_♀_ = 0.43, *p*_♀_ = 0.004, *r*_♂_ = 0.36, *p*_♂_ = 0.001) correlated with age.

The correlations observed, in particular the relationship between sense of coherence and both connectedness to nature and age, raised the question of the existence of a cause and effect relationship. Therefore, we performed a multiple regression analysis with these three variables as explanatory variables and the importance attributed to particular objectives as the response variable (Table 3).

As can be seen, the fashion objective explained the most variance in the group of women. The importance of this goal was very strongly associated with sense of comprehensibility. Feeling emotionally connected to the natural world was important for the physical fitness objective for both sexes.

Additionally, indicator variables in regression were used to examine differences between the sexes regarding how much the explanatory variables were related to (β) evaluating objectives. There was a statistically significant difference concerning sense of meaningfulness; its relationship with the fashion motivation was stronger among women and negative in character (*t* = 2.73, *p* = 0.007). There was also a difference in the relationship of sense of meaningfulness with boosting confidence and gaining appreciation from others. In women this relationship was negative, while in men it was positive, though statistically insignificant (*t* = 2.46, *p* = 0.016). A similar difference was observed in how this dimension of sense of coherence related to fulfilling the need for activity (*t* = 2.13, *p* = 0.036).

The last analysis was the multiple regression with age, sense of coherence, and feeling emotionally connected to the natural world as explanatory variables. The response variables were the values attributed to individual dimensions of the motivational function of the objective (Table 4).

The multiple regression analysis showed that motivational value among women was positively related to sense of meaningfulness and negatively to sense of comprehensibility. On the other hand, among men it was positively related to sense of manageability. In men, a positive relationship between connectedness to nature and time management and a negative relationship between age and time management was observed. Among women, age was negatively associated with persistence in action. For men, this variable was positively associated with sense of manageability. Sense of comprehensibility increased men’s motivational conflict, whereas in women this IPAO dimension was positively related to feeling emotionally connected to the natural world. Additionally, we used indicator variables in regression to examine intersexual differences in how much explanatory variables related to (β) the motivational function of the objective. The analysis showed one statistically significant difference: Feeling emotionally connected to the natural world was linked with motivational conflict positively in women and negatively in men (*t* = 2.26, *p* = 0.026).

## 4. Discussion

The goal of the presented study was to verify to what extent a sense of coherence and connectedness to nature (CN) are associated with the motivation to do karate and goals set by athletes. It was hypothesized that this relationship would be present in both men and women, but would be of a different character. Results indicated that these hypotheses were correct, but only with regard to some of the goals and their motivational function dimensions. Importantly for this work, connectedness to nature was positively associated with the sense of importance of goals, such as physical fitness, managing stress, and company of others. Undoubtedly, despite the relatively weak strength of these relationships, this is a result that indicates that having a sense of connectedness to the natural environment may be of importance for individuals who do karate and how they perceive the goals of their physical activity.

The results concerning the relationship between connectedness to nature and the importance of the managing stress goal was not surprising. Previous research suggested [49,50] that this was associated with a personality-related tendency toward conscious mindfulness, which, in turn, was also associated with higher ability to cope with stress. Many types of coping training are based on strengthening the skill of mindfulness. Thus, our result can be explained by the idea that those who practice karate and who have a stronger sense of belonging to the natural environment identify techniques that are effective regarding coping with stress in a more conscious manner, which is an important motivation for engaging in sports. However, it is unclear why this result was significant only for men; it is possible that reducing stress through taking part in martial arts is associated with men having a higher tendency to use active strategies for coping with stress [51].

Connectedness to nature was associated in both men and women with the importance of the physical fitness goal. To date, several studies have detailed a positive relationship between CN and psychological wellbeing [50] and engaging in intrinsic motivation [52]; our results suggest that among people who practice karate, the positive relationships between these variables are also connected with the importance of pursuing physical well-being. This is an important result, indicating that training in a natural environment, and the resulting increase in the sense of belonging to that environment, may lead to looking after one’s physical activity becoming an important, internally-motivated goal for an athlete.

CN was significantly related to the goals that motivate athletes to do karate. The strength of these relationships was small and they were present only in two dimensions of the motivational function of goals, motivational conflict and time management. A stronger sense of CN led to a greater sense of motivational conflict in women, whereas in men no such relationship was present. It can be inferred that the more someone feels attached to nature and wants to spend time in nature, the more any activity done away from nature is in conflict with important motivations. The fact that this relationship was not observed for men may be explained by the observed relationship between CN and time management. It may be that living in harmony with nature allows people who practice karate to better organize their time, so that the goals associated with doing sports are not in conflict with other life goals. Another way to explain these results would be in terms of the roles that men and women who participate in sports have in society. It might be the case that women play different roles in their lives, some of which are not fully realized [53]. The stronger the connection to the natural world, the harder it is to fulfill other goals. Men, however, very often decide to link their karate practice with work, for example as coaches [54].

Sense of coherence was shown to have a particular relationship with the motivational aspects of goals associated with doing karate. These relationships were shaped in different ways in men and in women. Women who do karate were more motivated if their sense of meaningfulness was higher, which was considered to be a motivational–emotional aspect. Interestingly, the cognitive aspect (of sense of comprehensibility) was negatively associated with the motivational value of the goals of karate. These results suggest that women who do karate were more eager to engage in sports if they feel that their life has meaning and it is worthwhile to engage in effort, while attempts to understand and process information coming from the environment decreased motivation. In men, the motivation to do karate was not associated with these dimensions of coherence, but this may depend on sense of manageability—progress on the path of karate is an important motivational aspect for these athletes.

It is worth stressing that every dimension of coherence, both in women and in men, was correlated with connectedness to nature. This result is in line with previous results of Cervinka, et al. [55], who reported a relationship between CN and mental health, well-being, and meaningfulness.

Results regarding the importance of goals associated with doing karate were in line with the results of research regarding goals of physical activity and, for example, extreme sports [56,57]. Among men, the main objective for practicing karate was physical fitness, followed by health and well-being; among women, the most important objectives were physical fitness, slim figure, and well-being. The objectives related to doing karate listed by male and female athletes seemed to be in line with those listed by people participating in physical activity [56]. Another study conducted on a Malaysian sample [58] of 703 men and 657 women showed that women, to a greater extent than men, were interested in the improvement of their looks and physical condition, whereas men were more interested in competition. Furthermore, one study on more than 1400 Polish women indicated that the main goal of women undertaking physical activity was to improve their physique and to maintain it, as well as the improvement and maintenance of physical fitness [59]. Aaltonen et al. [60] investigated the motivations of inactive and active individuals. For both the active and inactive participants, the most important motivation for undertaking physical activity was a desire to be in good physical condition and to improve their mental health. However, health and well-being were less frequent reasons for women undertaking physical activity. Karate involves “The Way”, therefore it is linked with “The Way” to well-being in life. The founder of Shotokan Karate, Gichin Funakoshi, said “*The ultimate goal of karate is not to win or lose, but improving the character of people who practice karate*”.

Interestingly, results for the motivational function of goals for practicing karate were relatively low when compared to the populational norms, especially on the time management and persistence in action scales, which may indicate that, despite the fact that the participants were experienced karate athletes, regular training and competitions did not constitute an aspect around which they organize their lives. A similar result was observed in individuals who do extreme sports (including extreme forms of martial arts) [57].

The main limitation of the current study concerns its cross-sectional nature and the fact that researched relationships cannot indicate causality. Apart from that, obtained results cannot be generalized to beginners because only advanced karateka were studied. The lack of elements of Eastern philosophy in other martial arts (e.g., MMA - mixed martial arts) prevents the generalization of these results to such arts. Another limitation is the small number of respondents; in future research, groups should be bigger. In future studies it would be good to use a measurement of mindfulness, which could be the link between motivation, coherence, and sense of connectedness to nature.

## 5. Conclusions

The main objective for practicing karate for men was physical fitness, followed by pleasure from physical activity and well-being; among women, the most important objectives were well-being, physical fitness, and having a slim figure. For both sexes, levels of connectedness to nature were highest when physical fitness was the primary motivation. In men, a positive impact of connectedness to nature and a negative impact of age on time management was observed. Feeling emotionally connected to the natural world affected motivational conflict positively in women and negatively in men. Understanding both the natural environment and the utility of setting sport-related goals led to increasing one’s efforts on the way to both successes and defeats, and, most of all, overcoming one’s weaknesses.

## Figures and Tables

**Table 1:** Importance of goals of practicing Karate.

Objective	♀		♂		*t*	*p*
	M	SD	M	SD		
Health	4.21	0.75	4.13	0.91	0.52	0.602
Physical Fitness	4.57	0.63	4.59	0.62	−0.14	0.887
Company of Others	3.48	1.02	3.56	1.10	−0.44	0.662
Fit, Shapely Body	4.31	0.78	3.87	1.03	2.43	0.016
Well-Being	4.62	0.66	4.41	0.78	1.48	0.140
Fashion	2.55	1.42	2.58	1.47	−0.11	0.916
Boosting Confidence	2.55	1.42	2.72	1.44	−0.63	0.531
Pleasure from Physical Activity	4.17	0.93	4.46	0.65	−2.06	0.042
Escape from Everyday Life	3.45	1.21	3.59	1.20	−0.60	0.551
Managing Stress	3.64	1.12	3.87	1.04	−1.13	0.261
Fulfilling the Need for Activity	3.81	0.92	3.99	0.91	−1.04	0.300
Promoting Physical Activity	3.10	1.23	2.94	1.36	0.62	0.536

**Table 2:** Mean results of sense of coherence and connectedness to nature.

Variables	♀		♂		*t*	*p*
	M	SD	M	SD		
SOC-29 Sense of Comprehensibility	45.48	6.18	46.66	7.92	−0.85	0.398
SOC-29 Sense of Manageability	49.24	7.17	48.28	8.92	0.60	0.547
SOC-29 Sense of Meaningfulness	42.93	6.17	40.62	7.71	1.69	0.094
Connectedness to Nature	47.64	5.79	47.01	6.64	0.53	0.600

**Table 3:** Multiple regression on the motivations for physical activity (response variability) and age, sense of coherence dimensions, and connectedness to nature (explanatory variables).

Objective	Sex	β [*p*]					% of Variance Explained, F, *p*
		Age	Comprehensibility	Manageability	Meaningfulness	Connectedness to Nature	Multiple Regression
Health	♀	0.27	−0.08	0.19	0.05	0.21	23%, 2.09, 0.089
	♂	0.19	0.00	−0.09	0.26	0.06	12%, 2.16, 0.067
Physical Fitness	♀	0.06	0.04	0.00	−0.13	**0.41 [0.025]**	15%, 1.30, 0.287
	♂	0.14	−0.06	0.08	0.14	**0.25 [0.031]**	**16%, 3.02, 0.015**
Company of Others	♀	−0.17	−0.11	0.20	−0.37	0.12	15%, 1.25, 0.308
	♂	−0.05	0.11	−0.05	0.16	**0.26 [0.029]**	**13%, 2.45, 0.041**
Fit, Shapely Body	♀	0.13	0.19	0.20	**–0.71 [0.008]**	0.16	19%, 1.66, 0.170
	♂	−0.18	0.03	−0.13	−0.05	0.22	7%, 1.27, 0.284
Well-Being	♀	−0.17	0.47	**–0.57 [0.038]**	0.31	0.26	23%, 2.15, 0.081
	♂	0.11	0.23	−0.08	0.02	0.05	9%, 1.46, 0.213
Fashion	♀	−0.10	**0.49 [0.043]**	0.25	**–0.90 [<0.001]**	0.11	**35%, 3.91, 0.006**
	♂	−0.25	0.14	−0.14	−0.14	0.10	10%, 1.72, 0.140
Boosting Confidence	♀	−0.09	0.36	−0.14	**–0.59 [0.022]**	0.30	23%, 2.16, 0.080
	♂	**–0.26 [0.035]**	0.00	**–0.43 [0.015]**	0.12	0.09	**19%, 3.63, 0.005**
Pleasure from Physical Activity	♀	−0.08	−0.22	0.25	0.45	−0.20	20%, 1.74, 0.150
	♂	−0.16	−0.02	−0.19	**0.57 [<0.001]**	0.14	**22%, 4.54, 0.001**
Escape from Everyday Life	♀	−0.29	0.22	−0.18	−0.02	0.15	8%, 0.64, 0.670
		0.05	−0.05	−0.07	−0.02	0.19	4%, 0.58, 0.717
Managing Stress	♀	−0.13	**–0.65 [0.015]**	0.31	0.28	0.18	23%, 2.13, 0.084
	♂	**0.27 [0.031]**	−0.12	−0.14	−0.13	**0.31 [0.007]**	**17%, 3.12, 0.013**
Fulfilling the Need for Activity	♀	−0.19	0.07	−0.25	**0.51 [0.050]**	0.14	18%, 1.61, 0.181
	♂	0.18	0.00	0.29	−0.10	0.21	**17%, 3.33, 0.009**
Physical Activity Promotion	♀	−0.08	−0.24	0.36	0.04	0.15	11%, 0.84, 0.524
	♂	−0.15	−0.06	0.22	−0.06	0.20	9%, 1.59, 0.174

*Note.* ♂ and ♀ denote male and female participants, respectively. For age, comprehensibility, manageability, meaningfulness, and connectedness to nature, *p* values are given only when <0.05. Throughout the table, boldface indicates cells with *p* < 0.05.

**Table 4:** Multiple regression with the motivational function of the objective (response variable) and age, sense of coherence, and connectedness to nature (explanatory variables).

Objectives	Sex	β [*p*]					% of Variance Explained, F, *p*
		Age	Comprehensibility	Manageability	Meaningfulness	Connectedness to Nature	Multiple Regression
Motivational Value	♀	−0.10	**–0.49 [0.050]**	−0.01	**0.49 [0.050]**	0.24	23%, 2.17, 0.079
	♂	−0.12	−0.08	**0.39 [0.029]**	0.10	0.04	**18%, 3.52, 0.006**
Time Management	♀	−0.04	−0.46	0.14	0.15	0.26	14%, 1.17, 0.345
	♂	**–0.28 [0.027]**	0.09	−0.15	0.12	**0.28 [0.017]**	12%, 2.24, 0.058
Persistence in Action	♀	**–0.40 [0.030]**	−0.03	−0.14	0.27	−0.17	15%, 1.25, 0.308
	♂	−0.10	−0.22	**0.45 [0.012]**	0.11	−0.16	**17%, 3.33, 0.009**
Motivational Conflict	♀	−0.04	0.26	−0.24	−0.04	**0.37 [0.044]**	15%, 1.23, 0.315
	♂	0.05	**0.32 [0.050]**	−0.11	−0.04	−0.13	7%, 1.25, 0.293

*Note.* ♂ and ♀ denote male and female participants, respectively. For age, comprehensibility, manageability, meaningfulness, and connectedness to nature, *p* values are given only when <0.05. Throughout the table, boldface indicates cells with *p* < 0.05.

## References

[1] Pretty J., Rogerson M., Barton J. (2017). Green Mind Theory: How Brain-Body-Behaviour Links into Natural and Social Environments for Healthy Habits. Int. J. Environ. Res. Public Health.

[2] Mantler A., Logan A.C. (2015). Natural environments and mental health. Adv. Integr. Med..

[3] Andersen L.B., Mota J., Di Pietro L. (2016). Update on the global pandemic of physical inactivity. Lancet.

[4] Pasek M., Ziółkowski A. (2014). Ekologiczny Wymiar Kultury Fizycznej. [Ecological Aspect of Physical Culture].

[5] McMahan E.A., Estes D. (2015). The effect of contact with natural environments on positive and negative affect: A meta-analysis. J. Posit. Psychol..

[6] Lubans D., Richards J., Hillman C., Faulkner G., Beauchamp M., Nilsson M., Kelly P., Smith J., Raine L., Biddle S. (2016). Physical activity for cognitive and mental health in youth: A systematic review of mechanisms. Pediatrics.

[7] Williams J.M.G., Kabat-Zinn J. (2011). Mindfulness: Diverse perspectives on its meaning, origins, and multiple applications at the intersection of science and dharma. Contemp. Buddhism.

[8] Mayer F.S., Frantz C.M. (2004). The connectedness to nature scale: A measure of individuals’ feeling in community with nature. J. Environ. Psychol..

[9] Cox J.C. (1993). Traditional Asian Martial Arts Training: A Review. Quest.

[10] Tomas J., Saragoça J. (2018). Budo as philosophical background of Karate-Do: Does the training method really matter?. Ido Movement for Culture. J. Martial Arts Anthropol..

[11] Gladwell V.F., Brown D.K., Wood C., Sandercock G.R., Barton J.L. (2013). The great outdoors: How a green exercise environment can benefit all. Extrem. Physiol. Med..

[12] Triguero-Mas M., Donaire-Gonzalez D., Seto E., Valentin A., Martinez D., Smith G., Hurst G., Carrasco-Turigas G., Masterson D., van den Berg M. (2017). Natural outdoor environments and mental health: Stress as a possible mechanism. Environ. Res..

[13] Calogiuri G., Chroni S. (2014). The impact of the natural environment on the promotion of active living: An integrative systematic review. BMC Public Health.

[14] Irvine K.N., Warber S.L. (2002). Greening healthcare: Practicing as if the natural environment really mattered. Altern. Ther. Health Med..

[15] Wilczenski F.L., Cook A.L. (2014). Toward positive and systemic mental health practices in schools: Fostering social-emotional learning through service. Health Psychol. Rep..

[16] Mitchell R. (2013). Is physical activity in natural environments better for mental health than physical activity in other environments?. Soc. Sci. Med..

[17] Koźlak W. (2012). Rola zajęć terenowych w kształtowaniu świadomości i postaw proekologicznych dzieci ze szczególnym uwzględnieniem wycieczek i ścieżek dydaktycznych [A role of outdoor lessons in shaping knowledge and proecological attitudes of children with special consideration of trips and didactic paths]. Ekologia.

[18] Groenewegen P.P., van den Berg A.E., de Vries S., Verheij R.A. (2006). Vitamin G: Effects of green space on health, well-being, and social safety. BMC Public Health.

[19] Pasek M. (2013). Postawy Prosomatyczne Uczniów Jako Efekt Zajęć z Wychowania Fizycznego w Terenie i w Sali w Świetle Wybranych Uwarunkowań Osobniczych i Środowiskowych [Prosomatic Attitudes of Students as the Effect of Outdoor and Indoor Physical Education Lessons in the Light of Chosen Personal and Environmental Conditionings].

[20] Hartig T., Evans G.W., Jamner L.D., Davis D.S., Gärling T. (2003). Tracking restoration in natural and urban field settings. J. Environ. Psychol..

[21] Kuo F.E., Sullivan W.C. (2001). Aggression and Violence in the Inner City: Effects of Environment via Mental Fatigue. Environ. Behav..

[22] Mayer F.S., Frantz C.M., Bruehlman-Senecal E., Dolliver K. (2009). Why is nature beneficial?: The role of connectedness to nature. Environ. Behav..

[23] Hinds J., Sparks P. (2008). Engaging with the natural environment: The role of affective connection and identity. J. Environ. Psychol..

[24] Shepard C.L., Speelman L.R. (1986). Affecting Environmental Attitudes through Outdoor Education. J. Environ. Educ..

[25] Thompson Coon J., Boddy K., Stein K., Whear R., Barton J., Depledge M.H. (2011). Does participating in physical activity in outdoor natural environments have a greater effect on physical and mental wellbeing than physical activity indoors? A systematic review. Environ. Sci. Technol..

[26] Kerr J., Sallis J.F., Saelens B.E., Cain K.L., Conway T.L., Frank L.D., King A.C. (2012). Outdoor physical activity and self rated health in older adults living in two regions of the U.S. Int. J. Behav. Nutr. Phys. Act..

[27] Pasek M., Pieńkos K. (2004). Próba wieloczynnikowej waloryzacji lasów dla potrzeb sportu i turystycznych form rekreacji [Attempt of multifactor valuation of forests for sport and touristic forms of recreation]. Problemy Zrównoważonego Rozwoju Turystyki, Rekreacji i Sportu w Lasach [Problems of Balanced Development of Tourism, Recreation and Sport in the Forests].

[28] Lipowski M., Bulinski L., Krawczynski M. (2009). Physical activities among other types of health-related behaviour in people losing weight. Med. Sci. Monit..

[29] Kilpatrick M., Hebert E., Bartholomew J. (2005). College students’ motivation for physical activity: Differentiating men’s and women’s motives for sport participation and exercise. J. Am. Coll. Health.

[30] Zaggelidis G., Martinidis K., Zaggelidis S. (2004). Comparative study of factors–motives in beginning practicing judo and karate. Phys. Train. Fit. Combat..

[31] Stefanek K.A. (2004). An Exploration of Participation Motives among Collegiate Taekwondo Participants. Ph.D. Thesis.

[32] Jones G.W., Mackay K.S., Peters D.M. (2006). Participation motivation in martial artists in the west midlands region of England. J. Am. Coll. Health.

[33] Twemlow S.W., Lerma B.H., Twemlow S.W. (1996). An analysis of students’ reasons for studying martial arts. Percept. Mot. Skills.

[34] Toskovic N.N. (2001). Alterations in selected measures of mood with a single bout of dynamic Taekwondo exercise in college-age students. Percept. Mot. Skills.

[35] Madden M. (1990). Attributions of Control and Vulnerability at the Beginning and End of a Karate Course. Percept. Mot. Skills.

[36] Lu C. (2003). An understanding of body-mind relation based on Eastern movement disciplines and its implication in physical education. Avante.

[37] Jakubowska H., Channon A., Matthews C.R. (2016). Gender, Media, and Mixed Martial Arts in Poland. J. Sport Soc. Issues.

[38] Van Tuyckom C., Scheerder J., Bracke P. (2010). Gender and age inequalities in regular sports participation: A cross-national study of 25 European countries. J. Sports Sci..

[39] Bronikowski M., Laudanska-Krzeminska I., Tomczak M., Morina B. (2017). Sense of coherence, physical activity and its associations with gender and age among Kosovar adolescents: A cross-sectional study. J. Sports Med. Phys. Fit..

[40] Chalabaev A., Sarrazin P., Fontayne P., Boiché J., Clément-Guillotin C. (2013). The influence of sex stereotypes and gender roles on participation and performance in sport and exercise: Review and future directions. Psychol. Sport Exerc..

[41] Larsson G., Kallenberg K.O. (1999). Sense of coherence, socioeconomic conditions and health. Interrelationships in a nation-wide Swedish sample. Eur. J. Public Health.

[42] Langius A., Bjorvell H. (1993). Coping ability and functional status in a Swedish population sample. Scand. J. Caring Sci..

[43] Antonovsky A., Matarazzo J.D. (1984). The Sense of Coherence as a Determinant in Health. Behavioral Health: A Handbook of Health Enhancement and Disease Prevention.

[44] Lipowski M., Zaleski Z. (2015). Inventory of Physical Activity Objectives—A new method of measuring motives for physical activity and sport. Health Psychol. Rep..

[45] Antonovsky A. (1993). The structure and properties of the sense of coherence scale. Soc. Sci. Med..

[46] Antonovsky A. (1983). The sense of coherence: Development of a research instrument. Newsl. Res. Rep..

[47] Koniarek J., Dudek B., Makowska Z. (1993). Kwestionariusz Orientacji Życiowej. Adaptacja The Sense of Coherence Questionnaire (SOC) A. Antonovsky‘ego [Questionnaire of Life Orientation. Adaptation of Antonovsky’s: The Sense of Coherence Questionnaire]. Przegl. Psychol..

[48] Pasikowski T., Sęk H., Pasikowski T. (2001). Kwestionariusz poczucia koherencji dla dorosłych (SOC-29) [Sense of Coherence in Adults Questionnaire (SOC-29)]. Zdrowie-Stres-Zasoby. O Znaczeniu Poczucia Koherencji dla Zdrowia [Health-Stress-Resources. Sense of Coherence and Its Importance to Health].

[49] Wolsko C., Lindberg K. (2013). Experiencing connection with nature: The matrix of psychological well-being, mindfulness, and outdoor recreation. Ecopsychology.

[50] Howell A.J., Dopko R.L., Passmore H.A., Buro K. (2011). Nature connectedness: Associations with well-being and mindfulness. Pers. Individ. Differ..

[51] Anshel M.H., Sutarso T., Jubenville C. (2009). Racial and gender differences on sources of acute stress and coping style among competitive athletes. J. Soc. Psychol..

[52] Weinstein N., Przybylski A.K., Ryan R.M. (2009). Can nature make us more caring? Effects of immersion in nature on intrinsic aspirations and generosity. Pers. Soc. Psychol. Bull..

[53] Brannon L. (2005). Gender: Psychological Perspectives.

[54] Lance L.M. (2004). Gender Differences in Perceived Role Conflict Among University Student-Athletes. Coll. Stud. J..

[55] Cervinka R., Röderer K., Hefler E. (2012). Are nature lovers happy? On various indicators of well-being and connectedness with nature. J. Health Psychol..

[56] Lipowski M., Ussorowska A. (2018). The motivational function of an objective in physical activity and sport. Curr. Issues Pers. Psychol..

[57] Krokosz D. (2016). Uwarunkowania I Motywy Uczestnictwa w Sportach Ekstremalnych a Poczucie Zadowolenia z Życia [Determinants and Motives of Involvement in Extreme Sports in Regard to Satisfaction with Life]. Ph.D. Thesis.

[58] Molanorouzi K., Khoo S., Morris T. (2015). Motives for adult participation in physical activity: Type of activity, age, and gender. BMC Public Health.

[59] Lipowski M. (2006). Rekreacja Ruchowa Kobiet Jako Zachowanie Prozdrowotne—Uwarunkowania a Motywy Uczestnictwa [Physical Activity of Women as Pro-Health Behaviour—Determinants and Motives of Participation].

[60] Aaltonen S., Rottensteiner M., Kaprio J., Kujala U. (2014). Motives for physical activity among active and inactive persons in their mid-30 s. Scand. J. Med. Sci. Sports.

